# A Long-Term, Open-Label Safety and Tolerability Study of Lisdexamfetamine Dimesylate in Children Aged 4–5 Years with Attention-Deficit/Hyperactivity Disorder

**DOI:** 10.1089/cap.2021.0138

**Published:** 2022-03-15

**Authors:** Ann C. Childress, Eric Lloyd, Steven A. Johnson, Lhanoo Gunawardhana, Valerie Arnold

**Affiliations:** ^1^ Center for Psychiatry and Behavioral Medicine, Las Vegas, Nevada, USA.; ^2^ Takeda Pharmaceuticals USA, Inc., Bannockburn, Illinois, USA.; ^3^ Takeda Pharmaceuticals USA, Inc., Lexington, Massachusetts, USA.; ^4^ CNS Healthcare, Memphis, Tennessee, USA.

**Keywords:** attention-deficit/hyperactivity disorder, lisdexamfetamine dimesylate, preschool-aged children, safety, tolerability, efficacy

## Abstract

**Objective:**

To evaluate the long-term safety and tolerability of lisdexamfetamine dimesylate (LDX) in preschool-aged children (4–5 years of age inclusive) diagnosed with attention-deficit/hyperactivity disorder (ADHD).

**Methods:**

This phase 3 open-label study (ClinicalTrials.gov registry: NCT02466386) enrolled children aged 4–5 years meeting *Diagnostic and Statistical Manual of Mental Disorders, Fourth Edition, Text Revision* (*DSM-IV-TR*) criteria for a primary ADHD diagnosis and having baseline ADHD Rating Scale-IV Preschool version total scores (ADHD-RS-IV-PS-TS) ≥24 for girls or ≥28 for boys and baseline Clinical Global Impressions–Severity scores ≥4. Participants were directly enrolled or enrolled after completing one of two antecedent short-term LDX studies. Over 52 weeks of treatment, participants received once-daily dose-optimized LDX (5–30 mg). Safety and tolerability assessments included treatment-emergent adverse events (TEAEs) and vital sign changes. Clinical outcomes included ADHD-RS-IV-PS-TS changes from baseline.

**Results:**

Among 113 participants in the safety set, optimized LDX dose was 5, 10, 15, 20, and 30 mg in 1 (0.9%), 12 (10.6%), 21 (18.6%), 26 (23.0%), and 53 (46.9%) participants, respectively. Of the safety set, 69 participants (61.1%) completed the study. TEAEs were reported in 76.1% of participants; no serious TEAEs were reported. Only one type of TEAE was reported in >10% of participants (decreased appetite, 15.9%). Mean ± standard deviation (SD) changes in vital signs and body weight from baseline to week 52/or early termination (ET; *n* = 101) were 1.9 ± 7.73 mmHg for systolic blood pressure, 3.1 ± 7.58 mmHg for diastolic blood pressure, 4.7 ± 11.00 bpm for pulse, and 0.6 ± 1.38 kg for body weight. Over the course of the study, mean ± SD change in ADHD-RS-IV-PS-TS from baseline to week 52/ET was −24.2 ± 13.34 (*n* = 87).

**Conclusions:**

In this long-term 52-week study of children aged 4–5 years with ADHD, dose-optimized LDX (5–30 mg) was well tolerated and associated with reductions from baseline in ADHD symptoms.

## Introduction

Results from the 2016 National Survey of Children's Health (NSCH) indicated that ∼6.1 million children aged 2–17 years in the United States had ever received an attention-deficit/hyperactivity disorder (ADHD) diagnosis from a health care provider, including 388,000 children aged 2–5 years (Danielson et al. 2018). Pharmacologic interventions are recommended in children with ADHD whose symptoms do not improve with parent training and behavior management (Wolraich et al. 2019).

Psychostimulants are the recommended pharmacotherapy for children and adolescents diagnosed with ADHD (Wolraich et al. 2019). Although most ADHD pharmacotherapies are not approved for use in preschool-aged children by the U.S. Food and Drug Administration (FDA), pharmacotherapy has been used to treat ADHD in children <6 years of age (Visser et al. 2016; Danielson et al. 2018; Davis et al. 2019). Approximately 18% of children with current ADHD aged 2–5 years were prescribed ADHD pharmacotherapy in 2016 according to NSCH data, with most of these individuals aged 4–5 years (Danielson et al. 2018).

Lisdexamfetamine dimesylate (LDX) is approved in the United States for use in individuals aged ≥6 years diagnosed with ADHD (Vyvanse^®^ 2017). Treatment with LDX (30–70 mg) was more effective compared with placebo in treating ADHD symptoms and had a favorable short-term (4–7 weeks) safety and tolerability profile in children and adolescents (Biederman et al. 2007; Findling et al. 2011; Coghill et al. 2013). Two completed antecedent short-term LDX treatment studies examined lower doses in preschool-aged (4–5 years) children diagnosed with ADHD in support of a pediatric written request by the FDA.

In a phase 2 open-label study (ClinicalTrials.gov registry: NCT02402166) in children with ADHD aged 4–5 years, LDX was well tolerated with a starting dose of 5 mg uptitrated to a maximum dose of 30 mg (Childress et al. 2020a). After an 8-week treatment period, the most frequently reported treatment-emergent adverse events (TEAEs) were decreased appetite, insomnia, and upper respiratory tract infection (Childress et al. 2020a). A 26-point mean reduction from baseline in the ADHD Rating Scale-IV Preschool version total scores (ADHD-RS-IV-PS-TS) was observed at the final on-treatment visit, and the majority (83%) of the study participants showed improvement on the Clinical Global Impressions–Improvement (CGI-I) scale (Childress et al. 2020a).

In a phase 3, placebo-controlled, fixed-dose, short-term, 6-week study (NCT03260205) of LDX (5, 10, 20, or 30 mg) or placebo in children aged 4–5 years with ADHD, LDX was more efficacious than placebo in reducing symptoms and had a safety and tolerability profile consistent with previous LDX studies in older children (Childress et al. 2020b).

This article reports the findings from a 52-week phase 3 study (NCT02466386) that further examined the long-term safety and tolerability of LDX (5–30 mg) in preschool-aged children (4–5 years of age inclusive) diagnosed with ADHD.

## Methods

### Study design

**FIG. 1.**
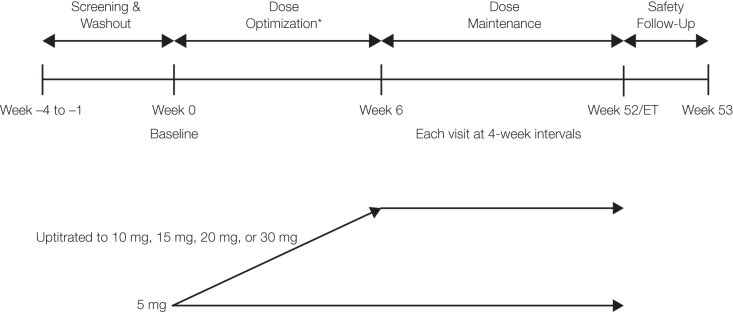
Study design. *All participants underwent the dose optimization period except those who enrolled following the phase 2 antecedent study, which included a similar dose optimization phase. Week 52/ET, data from protocol-defined last treatment study visit or early termination visit.

This was a phase 3, open-label multicenter study with participants who were directly enrolled or enrolled after completing one of two antecedent short-term LDX studies (phase 2, NCT02402166 or phase 3, NCT03260205) (Childress et al. 2020a, 2020b). This long-term (52-week) study included four periods: screening and washout, dose optimization, dose maintenance, and safety follow-up (Fig. 1).

The study was conducted in accordance with guidelines of the International Council for Harmonisation Good Clinical Practice and the principles of the Declaration of Helsinki, as well as other applicable local ethical and legal requirements. Signed informed consent and assent of the study participant and the participant's parent(s) or legally authorized representative (LAR) were required before any study-related procedures, including screening assessments. The study protocol, protocol amendments, final approved informed consent and assent documents, and all relevant supporting information were submitted by the investigator to the institutional review board (IRB) and approved by the IRB and regulatory agency (as appropriate) before study initiation.

### Participants

The study enrolled boys and girls aged 4–5 years meeting *Diagnostic and Statistical Manual of Mental Disorders, Fourth Edition, Text Revision* (*DSM-IV-TR*) criteria for a primary ADHD diagnosis (American Psychiatric Association 2000). Participants were required to have baseline scores ≥28 (boys) or ≥24 (girls) on the ADHD-RS-IV-PS-TS and ≥4 on the Clinical Global Impressions–Severity (CGI-S) scale.

The participants were also required to have undergone an adequate course of nonpharmacologic treatment or have symptoms severe enough to warrant enrollment without prior nonpharmacologic treatment, be engaged in a structured group activity that allowed for assessment of ADHD symptoms and impairment outside of the home (e.g., preschool, sports, Sunday school), have a screening Peabody Picture Vocabulary Test standard score ≥70, and have lived with the same parent/LAR for ≥6 months. The participants and their parents/LARs were also required to be willing and able to comply and be available with all testing and protocol requirements, including oversight of morning dosing.

Participants were excluded from the study if they were terminated from an antecedent LDX study for noncompliance, experienced a serious adverse event (SAE) or adverse event (AE) resulting in termination, or required or anticipated the need to take medications that have central nervous system effects or affect performance, such as sedating antihistamines and decongestant sympathomimetics or monoamine oxidase inhibitors.

Participants were also excluded if they had any concurrent or acute illness, condition, or disability that could confound safety assessments or increase participant risk or had a current controlled or uncontrolled comorbid Axis I or II psychiatric disorder (e.g., posttraumatic stress disorder, adjustment disorder, bipolar disorder, pervasive development disorder, obsessive-compulsive disorder, psychosis/schizophrenia). Additional exclusion criteria included a history of serious cardiac problems; a screening or baseline blood pressure (BP) ≥95th percentile for age, sex, and height; previous failure to fully respond to amphetamine therapy; and a documented allergy, hypersensitivity, or intolerance to amphetamine or LDX excipients.

### Treatment

All participants underwent a dose optimization period at the start of the study, except for those who enrolled following the phase 2 antecedent study, which included a similar dose optimization phase. During the dose optimization period, participants received a once-daily morning dose of 5, 10, 15, 20, or 30 mg of LDX, with a beginning dose of 5 mg and stepwise uptitration until an optimal dose was reached. The dose optimization was performed during the first 6 weeks to ensure that participants received the optimal dosage of the study drug based on TEAEs and clinical criteria.

The participants' responses during the dose optimization period were divided into one of three categories: (1) intolerable response, with participants experiencing intolerable AEs; (2) ineffective response, where the participants failed to achieve at least a 30% reduction in ADHD-RS-IV-PS-TS from baseline of the antecedent study (if applicable) and a CGI-I score of 1 or 2; and (3) acceptable response, where the participants achieved at least a 30% reduction in ADHD-RS-IV-PS-TS from baseline of the antecedent study (if applicable) and a CGI-I score of 1 or 2 with tolerable AEs.

Participants assessed as having an intolerable response were tapered to a lower LDX dose. If the lower dose also produced intolerable side effects, the participant was discontinued from the study. Participants assessed as having an ineffective response were titrated to the next highest LDX dose if available, provided no tolerability issues arose. Dose optimization continued until an acceptable response was achieved. Participants assessed as having an acceptable response were maintained on their current dose for the remainder of the study.

### End points

Safety and tolerability assessments included TEAEs and changes in vital signs, body weight, and body mass index (BMI); 12-lead electrocardiogram (ECG) recordings; sleep assessments (Children's Sleep Habits Questionnaire [CSHQ] and sleep diary); and the Columbia-Suicide Severity Rating Scale (C-SSRS) (Owens et al. 2000; Posner et al. 2011).

An AE was defined as any untoward medical occurrence in a clinical investigation subject administered a pharmaceutical product and that does not necessarily have a causal relationship with this treatment. An SAE was defined as any untoward event resulting in death, life-threatening condition, inpatient hospitalization or prolongation of existing hospitalization, persistent or significant disability/incapacity, congenital abnormality/birth defect, or important medical event (e.g., allergic bronchospasm, blood dyscrasias or convulsions, or development of drug dependence or drug abuse). A severe AE was defined as an event that interrupted usual activities of daily living, significantly affected clinical status, or may have required intensive therapeutic intervention.

A physical examination was performed at screening and baseline by a qualified licensed individual (physician, physician assistant, or nurse practitioner). In addition, an abbreviated physical examination was required before the baseline visit if >30 days had elapsed since the screening visit. Vital signs (systolic blood pressure [SBP], diastolic blood pressure [DBP], and pulse), weight, and 12-lead ECGs were assessed at screening, baseline, and each on-treatment visit. SBP and DBP measurements (sitting) were performed at each visit to the site. The vital sign measurements (BP, pulse, respiratory rate, and ECG) were obtained after the participant had rested for a minimum of 5 minutes. Any significant deviation of vital sign measurement from baseline was recorded as an AE by the investigator. Body weight was measured at screening, baseline, and each on-treatment visit.

Sleep was assessed at screening, baseline, and each on-treatment visit with the CSHQ and sleep diary. The CSHQ is a 33-item parent-/LAR-reported questionnaire that evaluates common sleep problems in children. It is grouped into eight subscales (bedtime resistance, sleep-onset delay, sleep duration, sleep anxiety, night awakenings, parasomnias, sleep-disordered breathing, and daytime sleepiness) based on the participant's sleep behavior. A sleep diary was completed by the participant's parent/LAR to log daytime napping, bedtime, and wake time.

The C-SSRS (pediatric/cognitively impaired version) was administered at screening, baseline, and each on-treatment visit, with the “lifetime recent” version completed at screening and the “since last visit” version completed at postscreening visits. The C-SSRS is a semistructured interview that captures the occurrence, severity, and frequency of suicide-related thoughts and behaviors during the study period. The interview included definitions and age-appropriate suggested questions to extract and analyze the type of information required to assess a suicide-related thought or behavior occurring during the course of the assessment period.

The efficacy end point was the change from baseline in clinician-administered ADHD-RS-IV-PS-TS at visit 1 and at each subsequent visit up to and including the end-of-study visit to capture the ADHD symptoms within each study period. The ADHD-RS-IV-PS is an 18-item clinician-administered instrument that rates ADHD symptom frequency defined by *DSM-IV-TR* criteria using examples appropriate for the developmental level of preschool children. The items are scored on a 4-point scale (range, 0 [never or rarely] to 3 [very often]); total score ranges from 0 to 54. The normative score is 13.9 for boys and 7.8 for girls (McGoey et al. 2007). The items can be further grouped into two 9-item subscales to assess inattention and hyperactivity/impulsivity. The ADHD-RS-IV-PS was used to guide dosing decisions and reviewed/completed by the investigator or subinvestigator.

The additional efficacy end point was the global evaluation of participant disease severity and improvement over time as measured by the CGI scale (Guy 1976). The severity of the participant's condition was assessed by the CGI-S, a 7-point scale ranging from 1 (normal, not at all ill) to 7 (among the most extremely ill subjects) at baseline of the antecedent study or at baseline (visit 0) of this study for the directly enrolled participants. The CGI-I assessed ADHD improvement (from the appropriate baseline visit) at each visit from visit 1 to the end-of-study visit or early termination (ET) visit. CGI-I was graded on a 7-point scale (range, 1 [very much improved] to 7 [very much worse]). The CGI-S and CGI-I were completed by a clinician trained and experienced in the evaluation of preschool children with ADHD. The CGI-I was used to guide dosing decisions and reviewed/completed by the principal investigator or subinvestigator.

The general cognitive ability of the participants was assessed by the Peabody Picture Vocabulary Test, Fourth Edition. It measures an individual's receptive (hearing) vocabulary for Standard American English and provides a quick estimate of verbal ability or scholastic aptitude. The Peabody Picture Vocabulary Test was administered by site personnel with training and experience in general psychological testing approved by the sponsor or delegated vendor.

### Data and statistical analysis

The safety analysis set consisted of all participants who took ≥1 dose of investigational product. The full analysis set consisted of all participants in the safety analysis set who had ≥1 postdose ADHD-RS-IV-PS-TS assessment during the study. Unless otherwise specified, demographic and baseline characteristics were sourced from the antecedent studies or from case report forms for directly enrolled participants. All analyses were limited to descriptive statistics for observed data and change from baseline, where applicable.

Efficacy analyses were performed using the full analysis set. For all efficacy analyses, baseline was defined as either the baseline value from the antecedent study or, for directly enrolled participants, the last observation before the first dose of investigational product. There was no primary efficacy end point defined for this study.

Efficacy and safety data were summarized by optimized dose in a *post hoc* analysis. The optimized dose was established by the week 6 visit (or week 8 visit for patients who enrolled from the phase 2 antecedent study and did not undergo a dose optimization period in the current study; Fig. 1). For any participant who discontinued before week 6/8, the optimized dose was selected as the last dose level exposed. For any participant who changed dose after week 6/8, the optimized dose was set at the dose level the participant received with greatest frequency. In the summaries by optimized dose, participants were evaluated for a single dose level with all usable data regardless of the actual dose level at the time of the data point.

## Results

### Participant disposition and demographics

**FIG. 2.**
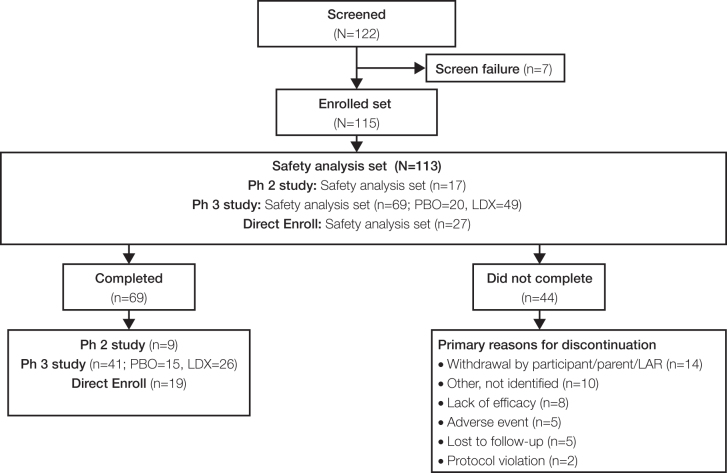
Participant disposition. Ph 2 (NCT02402166) and Ph 3 (NCT03260205) are antecedent studies. LAR, legally authorized representative; LDX, lisdexamfetamine dimesylate; PBO, placebo; Ph, phase.

Of the 122 participants screened, 115 were enrolled in the study. The safety analysis set had 113 participants who were either rollover participants completing antecedent studies (*n* = 86) or directly enrolled participants (*n* = 27; Fig. 2). A total of 69 participants (61.1%) from the safety set completed the study. The most frequently reported reasons for discontinuation from the study were withdrawal by the subject or parent(s)/LAR (*n* = 14) and lack of efficacy (*n* = 8).

The mean age, at the time of consent for study participation, was 4.8 ± 0.63 years (*n* = 113; Table 1). There were more boys (80/113 [70.8%]) than girls (33/113 [29.2%]), and the majority of participants were White (63/113 [55.8%]) or Black/African American (42/113 [37.2%]). Overall, the mean body weight and BMI were 20.9 ± 3.60 kg and 16.5 ± 2.00 kg/m^2^, respectively. Most participants were diagnosed with the combined ADHD subtype (106/113 [93.8%]) and had baseline CGI-S scores corresponding to “markedly ill” (66/113 [58.4%]). The mean Peabody Picture Vocabulary Test score was 102.1 ± 15.59.

**Table 1.** Participant Demographic and Baseline Clinical Characteristics, Safety Analysis Set

	Optimized LDX dose					Total (*N* = 113)
Characteristic	5 mg (*n* = 1)	10 mg (*n* = 12)	15 mg (*n* = 21)	20 mg (*n* = 26)	30 mg (*n* = 53)	
Antecedent study age (years),^a^ mean ± SD	5.0 (—)	4.7 ± 0.47	4.6 ± 0.51	4.6 ± 0.49	4.6 ± 0.49	4.6 ± 0.48
Current age (years),^b^ mean ± SD	5.0 (—)	4.8 ± 0.39	4.6 ± 0.59	4.7 ± 0.69	4.8 ± 0.67	4.8 ± 0.63
Male, *n* (%)	1 (100)	9 (75.0)	15 (71.4)	18 (69.2)	37 (69.8)	80 (70.8)
Race, *n* (%)						
White	1 (100)	7 (58.3)	13 (61.9)	16 (61.5)	26 (49.1)	63 (55.8)
Black/African American	0	4 (33.3)	7 (33.3)	9 (34.6)	22 (41.5)	42 (37.2)
American Indian/Alaska native	0	0	0	0	1 (1.9)	1 (0.9)
Multiple	0	1 (8.3)	0	0	1 (1.9)	2 (1.8)
Other	0	0	1 (4.8)	1 (3.8)	3 (5.7)	5 (4.4)
Weight (kg), mean ± SD	18.2 (—)	20.8 (1.51)	20.2 (2.64)	20.9 (2.46)	21.3 (4.61)	20.9 (3.60)
BMI (kg/m^2^),^c^ mean ± SD	15.0 (—)	16.3 (0.96)	16.5 (1.36)	16.3 (1.44)	16.6 (2.58)	16.5 (2.00)
ADHD-RS-IV-PS score, mean ± SD						
Total^d^	52.0 (—)	44.3 (6.77)	45.0 (7.47)	44.1 (6.90)	43.1 (5.99)	43.8 (6.54)
Inattention^d^	26.0 (—)	21.8 (4.53)	21.3 (4.14)	21.0 (4.90)	20.0 (3.69)	20.7 (4.18)
Hyperactivity/impulsivity^d^	26.0 (—)	22.4 (2.87)	23.7 (4.13)	23.1 (3.76)	23.1 (3.20)	23.2 (3.45)
ADHD subtype, *n* (%)						
Predominantly inattentive	0	0	2 (9.5)	0	0	2 (1.8)
Predominantly hyperactive/impulsive	0	1 (8.3)	0	0	3 (5.7)	4 (3.5)
Combined	1 (100)	11 (91.7)	19 (90.5)	26 (100)	49 (92.5)	106 (93.8)
Missing	0	0	0	0	1 (1.9)	1 (0.9)
Peabody Picture Vocabulary Test standard score, mean ± SD	112.0 (—)	96.3 ± 11.64	101.5 ± 16.41	102.3 ± 15.36	103.2 ± 16.34	102.1 ± 15.59
CGI-S score, mean ± SD	6.0 (—)	4.8 ± 0.72	5.0 ± 0.77	5.2 ± 0.54	4.9 ± 0.78	5.0 ± 0.73
CGI-S, *n* (%)						
Moderately ill	0	4 (33.3)	5 (23.8)	2 (7.7)	16 (30.2)	27 (23.9)
Markedly ill	0	6 (50.0)	12 (57.1)	18 (69.2)	30 (56.6)	66 (58.4)
Severely ill	1 (100)	2 (16.7)	3 (14.3)	6 (23.1)	4 (7.5)	16 (14.2)
Among the most extremely ill	0	0	1 (4.8)	0	3 (5.7)	4 (3.5)

Baseline was defined as the baseline value from the antecedent study (phase 2 study [NCT02402166]; phase 3 study [NCT03260205]) for antecedent participants or the last observation before the first dose of investigational product for directly enrolled participants.

^a^ Age was calculated as the difference between date of birth and date of informed consent for the antecedent study, truncated to years; *n* = 11 (10 mg); *n* = 16 (15 mg); *n* = 17 (20 mg); *n* = 41 (30 mg); *n* = 86 (total).

^b^ Current age was calculated as the difference between date of birth and date of informed consent for this study (NCT03260205), truncated to years.

^c^ BMI was calculated as [weight (kg)/height (m)^2^].

^d^ *n* = 20 (15 mg) and *n* = 112 (total).

ADHD, attention-deficit/hyperactivity disorder; ADHD-RS-IV-PS, ADHD Rating Scale-IV Preschool version; BMI, body mass index; CGI-S, Clinical Global Impressions–Severity; LDX, lisdexamfetamine dimesylate; SD, standard deviation.

### Drug exposure

Among the 113 participants in the safety analysis set, the optimized LDX dose was 5 mg in 1 participant (0.9%), 10 mg in 12 participants (10.6%), 15 mg in 21 participants (18.6%), 20 mg in 26 participants (23.0%), and 30 mg in 53 participants (46.9%). The median (range) duration of LDX exposure was 52 (1.9–55.6) weeks.

### Safety

As shown in Table 2, TEAEs were reported in 76.1% of participants; however, the majority were mild or moderate in severity. No serious TEAEs were reported, and the incidence of severe TEAEs was low (eight severe TEAEs reported in seven participants: decreased appetite [*n* = 2]; and sleep disorder, irritability, affect lability, influenza, crying, and neutropenia in one participant each). The only TEAE reported in >10% of participants was decreased appetite (15.9%). In the total population, 45.1% of participants had TEAEs that were considered related to the study drug according to the investigator. The frequency of TEAEs and severe TEAEs in the highest optimized LDX dose subgroup (LDX 30 mg) was similar to or lower than the frequency in lower optimized LDX dose subgroups.

**Table 2.** Summary of Treatment-Emergent Adverse Events, Safety Analysis Set

TEAE,* n *(%), m	Optimized LDX dose					Total (*N* = 113)
	5 mg (*n* = 1)	10 mg (*n* = 12)	15 mg (*n* = 21)	20 mg (*n* = 26)	30 mg (*n* = 53)	
Any TEAE	1 (100), 2	11 (91.7), 42	16 (76.2), 64	20 (76.9), 74	38 (71.7), 135	86 (76.1), 317
Related to study drug	0	6 (50.0), 8	11 (52.4), 22	14 (53.8), 23	20 (37.7), 39	51 (45.1), 92
Leading to discontinuation^a^	0	1 (8.3), 1	2 (9.5), 2	1 (3.8), 1	1 (1.9), 1	5 (4.4), 5
Severe^b^	0	1 (8.3), 1	3 (14.3), 3	0	3 (5.7), 4	7 (6.2), 8
Serious	0	0	0	0	0	0
TEAEs in ≥5% of total participants						
Decreased appetite	0	1 (8.3), 1	4 (19.0), 4	5 (19.2), 5	8 (15.1), 11	18 (15.9), 21
Pyrexia	0	3 (25.0), 3	2 (9.5), 2	5 (19.2), 6	1 (1.9), 1	11 (9.7), 12
Influenza	0	2 (16.7), 2	0	2 (7.7), 2	6 (11.3), 6	10 (8.8), 10
Pharyngitis streptococcal	0	1 (8.3), 3	3 (14.3), 3	1 (3.8), 1	4 (7.5), 4	9 (8.0), 11
Upper respiratory tract infection	0	1 (8.3), 1	2 (9.5), 4	1 (3.8), 1	4 (7.5), 9	8 (7.1), 15
Nasopharyngitis	0	1 (8.3), 1	3 (14.3), 4	1 (3.8), 2	3 (5.7), 4	8 (7.1), 11
Cough	0	1 (8.3), 2	1 (4.8), 1	2 (7.7), 3	3 (5.7), 8	7 (6.2), 14
Affect lability	0	0	3 (14.3), 4	1 (3.8), 1	3 (5.7), 3	7 (6.2), 8
Weight decreased	0	0	1 (4.8), 1	2 (7.7), 2	4 (7.5), 4	7 (6.2), 7
Initial insomnia	0	0	2 (9.5), 2	0	4 (7.5), 5	6 (5.3), 7
Vomiting	0	1 (8.3), 1	2 (9.5), 3	1 (3.8), 1	2 (3.8), 2	6 (5.3), 7

^a^ TEAEs leading to drug discontinuation: affect lability, *n* = 2; aggression, *n* = 1; mood swings, *n* = 1; decreased appetite, *n* = 1.

^b^ Severe TEAEs: decreased appetite, *n* = 2; sleep disorder, irritability, affect lability, influenza, crying, and neutropenia, *n* = 1 each.

LDX, lisdexamfetamine dimesylate; m, number of events; *n*, number of participants experiencing the event; TEAE, treatment-emergent adverse event.

Mean ± standard deviation (SD) changes from baseline to week 52 or ET (*n* = 101) were 4.67 ± 11.000 bpm for pulse, 1.92 ± 7.729 mmHg for SBP, 3.10 ± 7.581 mmHg for DBP, 0.56 ± 1.383 kg for body weight, and −18.65 ± 20.166 for BMI percentile (Table 3). Mean change from baseline in body weight and BMI by optimized dose is shown in Supplementary Figures S1 and S2. At week 52/ET, shifts from healthy weight (*n* = 6 [5.9%]) and overweight (*n* = 1 [1.0%]) categories to underweight were observed in seven participants from baseline. A total of 15 participants who were overweight at baseline shifted to a healthy weight category, and 7 participants who were obese at baseline shifted to either overweight (*n* = 3) or healthy weight (*n* = 4) categories. Three participants who were underweight at baseline shifted to overweight, and one participant shifted from overweight at baseline to obese at week 52/ET.

**Table 3.** Summary of Changes in Vital Signs, Body Weight, and Body Mass Index, Safety Analysis Set

Change from baseline*^a^* at week 52/ET, mean ± SD	Optimized LDX dose					Total (*N* = 113)
	5 mg (*n* = 1)	10 mg (*n* = 12)	15 mg (*n* = 21)	20 mg (*n* = 26)	30 mg (*n* = 53)	
Pulse,^b^ bpm	−11.97 (—)	6.52 ± 12.271	0.43 ± 12.274	5.33 ± 10.840	5.87 ± 10.033	4.67 ± 11.000
SBP,^b^ mmHg	9.63 (—)	0.99 ± 7.381	3.47 ± 10.692	3.62 ± 5.950	0.58 ± 7.206	1.92 ± 7.729
DBP,^b^ mmHg	7.70 (—)	5.03 ± 7.161	4.25 ± 8.239	4.13 ± 6.021	1.64 ± 8.082	3.10 ± 7.581
Weight,^b^ kg	1.90 (—)	1.47 ± 1.535	0.75 ± 1.169	0.47 ± 1.017	0.30 ± 1.507	0.56 ± 1.383
BMI percentile^b,c^	−9.92 (—)	−13.88 ± 12.594	−16.41 ± 17.249	−20.98 ± 19.104	−19.64 ± 23.250	−18.65 ± 20.166

^a^ Baseline is defined as the baseline value from the antecedent study (phase 2 study [NCT02402166]; phase 3 study [NCT03260205]) for antecedent participants or the last observation before the first dose of investigational product for directly enrolled participants.

^b^ *n* = 11 (10 mg), *n* = 18 (15 mg), *n* = 23 (20 mg), *n* = 48 (30 mg), *n* = 101 (total).

^c^ Percentiles were derived using the Centers for Disease Control and Prevention growth charts for children and adolescents.

BMI, body mass index; DBP, diastolic blood pressure; LDX, lisdexamfetamine dimesylate; SBP, systolic blood pressure; SD, standard deviation; week 52/ET, data from protocol-defined last treatment study visit or early termination visit.

No participant had a positive postbaseline C-SSRS response, and there were no reports of suicidal behavior or suicide attempts in any of the participants. Results from the CSHQ (Supplementary Table S1) and sleep diaries (Supplementary Table S2) show no notable overall trends across the optimized LDX dose subgroups.

### Efficacy

**FIG. 3.**
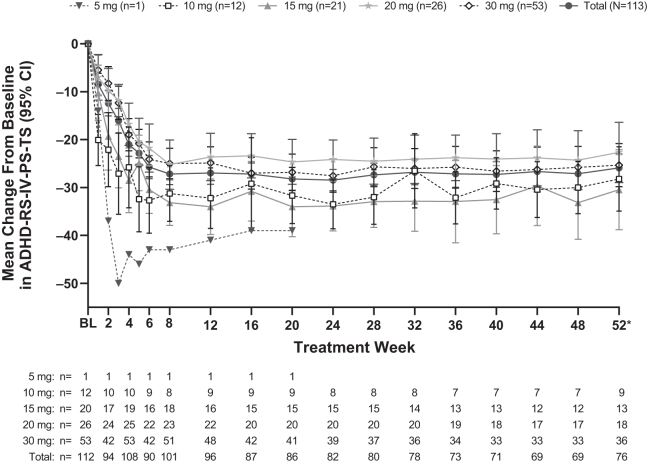
Mean change from BL in ADHD-RS-IV-PS-TS score by optimized LDX dose (safety analysis set). *Data from protocol-defined last treatment study visit or early termination visit. ADHD, attention-deficit/hyperactivity disorder; ADHD-RS-IV-PS-TS, ADHD Rating Scale-IV, Preschool version total scores; BL, baseline; LDX, lisdexamfetamine dimesylate.

**FIG. 4.**
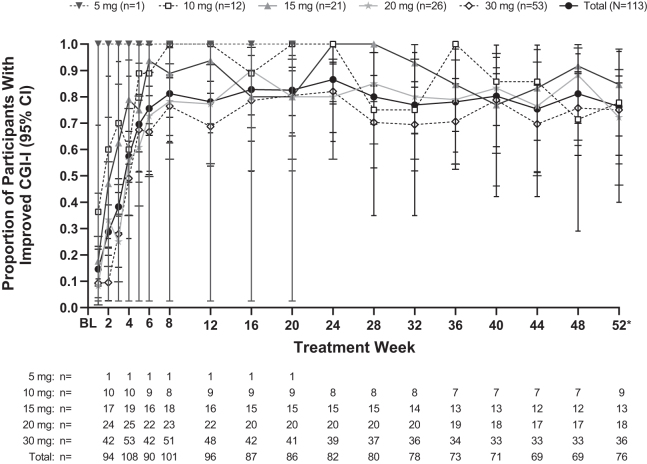
Proportion of participants with improved Clinical Global Impressions score by optimized LDX dose (safety analysis set). *Data from protocol-defined last treatment study visit or early termination visit. BL, baseline; CGI-I, Clinical Global Impressions–Improvement; LDX, lisdexamfetamine dimesylate.

Over the course of the study, the mean ± SD change in ADHD-RS-IV-PS-TS from baseline to week 52/ET was −24.2 ± 13.34, showing an overall decrease in ADHD symptoms (Fig. 3). Improvements in ADHD symptoms were also observed with the CGI-I scale, with 73.6% of participants having improved (very much improved [35.6%] or much improved [37.9%]) CGI-I measurements (Fig. 4). Similar trends in ADHD-RS-IV-PS-TS reduction and CGI-I scale improvement were observed across all optimized LDX dose subgroups (Figs. 3 and 4).

## Discussion

In this phase 3, open-label study, the long-term safety and tolerability of LDX (5–30 mg) was evaluated in children aged 4–5 years with ADHD. LDX was safe and well tolerated, with few TEAEs leading to withdrawal of study drug, and no SAEs or deaths associated with the investigational product. Overall, frequency of TEAEs and severe TEAEs did not increase with higher optimized LDX dose.

While the rates of TEAEs in this 52-week dose-optimized long-term study were comparable to the 8-week dose-optimized phase 2 antecedent LDX study conducted in preschool children (76.1% vs. 79%) (Childress et al. 2020a), they were higher than the prior 6-week, phase 3, fixed-dose antecedent study (76.1% vs. 46.6%) (Childress et al. 2020b). Decreased appetite was the most common TEAE (15.9% vs. 33% and 13.7% in the antecedent studies, respectively) (Childress et al. 2020b) and showed a possible dose-dependent trend in the current study (LDX 5 mg, 0%; LDX 10 mg, 8.3%; LDX 15 mg, 19.0%; LDX 20 mg, 19.2%; LDX 30 mg, 15.1%). However, this finding is limited by small sample sizes, particularly in the lower optimized LDX dose subgroups.

In this study, there were no clinically meaningful changes from baseline in pulse, ECG parameters, SBP, or DBP observed at week 52/ET. Variability in vital signs has been observed in previous pediatric LDX studies, with changes in pulse rate being the most consistent among them (Biederman et al. 2007; Findling et al. 2011; Coghill et al. 2013; Newcorn et al. 2017; Childress et al. 2020a, 2020b). In most studies, an increase in pulse rate was observed in pediatric populations treated with LDX (Biederman et al. 2007; Findling et al. 2011; Coghill et al. 2013; Newcorn et al. 2017). However, in the phase 2 antecedent study, a small decrease in pulse was observed (Childress et al. 2020a).

There were also no clinically meaningful changes in body weight or BMI in this study, although seven participants shifted to underweight from other weight categories at baseline. These findings are consistent with previous studies in preschool-aged children, older children, and adolescents on LDX treatment, which reported a small reduction in weight (Findling et al. 2011; Coghill et al. 2013; Newcorn et al. 2017; Childress et al. 2020a, 2020b). There were no notable trends in sleep based on the CSHQ and sleep diary assessments and no reports of suicidal behavior or suicide attempts, consistent with the prior 6-week, phase 3, fixed-dose antecedent study (Childress et al. 2020b).

Treatment with LDX (5–30 mg) reduced ADHD symptoms measured by ADHD-RS-IV-PS-TS from baseline to week 52/ET and improved ADHD symptoms measured by the CGI-I scale, both in the overall study population and in each of the optimized LDX dose subgroups. These results are consistent with recent studies of LDX in children with ADHD.

In the prior 6-week, phase 3, fixed-dose antecedent study, participants treated with LDX demonstrated a mean change from baseline in ADHD-RS-IV-PS-TS at week 6 of −14.7 versus −8.8 for the placebo cohort (Childress et al. 2020b). In a 4-week phase 3 study of older children aged 6–12 years, mean change from baseline in ADHD-RS-IV-PS-TS was −26.7 with LDX (fixed doses 30, 50, or 70 mg/d) and −6.2 with placebo (Biederman et al. 2007). Finally, a 7-week phase 3 study of children and adolescents aged 6–17 years reported a mean change from baseline in ADHD-RS-IV-PS-TS of −24.3 with LDX (30, 50, or 70 mg/d dose optimized) and −5.7 with placebo (Coghill et al. 2013; Childress et al. 2020a, 2020b).

Although this study is limited by the absence of a placebo control arm, this is in the best interests of the study participants because it is not recommended to keep participants with ADHD on placebo for the duration of a long-term study. Caution must be taken in interpreting results by optimized LDX dose subgroup because of the small sample sizes and potential impact of confounding factors that may affect the observations. Finally, there were limited data on Hispanic, Asian, and Native American populations, and psychiatric comorbidities were excluded, which may make it difficult to generalize.

## Conclusions

LDX at doses between 5 and 30 mg/d over 52 weeks of treatment was found to be safe and well tolerated in children aged 4–5 years with ADHD. No new safety signals were identified, and the efficacy profile was consistent with robust improvements in ADHD symptoms observed in previous studies of children, adolescents, and adults with ADHD (Biederman et al. 2007; Adler et al. 2008; Findling et al. 2011; Childress et al. 2020a, 2020b).

## Clinical Significance

LDX is approved for the treatment of ADHD in patients aged ≥6 years. Interest for clinical trial evidence of safety and efficacy for the treatment of younger children existed. This 52-week open-label study reports that LDX at doses between 5 and 30 mg/d in children aged 4–5 years with ADHD was found to be safe and well tolerated. No new safety signals were identified, and the efficacy profile was consistent with robust improvements in ADHD symptoms observed in previous studies of children, adolescents, and adults with ADHD.

## Data Sharing

The datasets, including the redacted study protocol, redacted statistical analysis plan, and individual participant's data supporting the results reported in this article, will be made available within three months from initial request, to researchers who provide a methodologically sound proposal. The data will be provided after its de-identification, in compliance with applicable privacy laws, data protection and requirements for consent and anonymization.

## Supplementary Material

Supplemental data

Supplemental data

Supplemental data

Supplemental data
