# Supplementary material for: A Long-Term, Open-Label Safety and Tolerability Study of Lisdexamfetamine Dimesylate in Children Aged 4–5 Years with Attention-Deficit/Hyperactivity Disorder
Source: J Child Adolesc Psychopharmacol. 2022 Mar 15;32(2):98–106. doi: 10.1089/cap.2021.0138 (PMC8971990; doi:10.1089/cap.2021.0138)
Supplement: Supplemental data [file Suppl_TableS1.docx]

# Supplementary Table 1. Children’s Sleep Habits Questionnaire Subscale Scores, Safety Analysis Set

|  | **Optimized LDX Dose** | | | | |  | |
| --- | --- | --- | --- | --- | --- | --- | --- |
| **Subscale score,**  **mean ± SD** | **5 mg**  **(n=1)** | **10 mg**  **(n=12)** | **15 mg**  **(n=21)** | **20 mg**  **(n=26)** | **30 mg**  **(n=53)** | | **Total**  **(N=113)** |
| Bedtime resistance |  |  |  |  |  | |  |
| Baseline^a^ | 15.0 (–) | 10.8±2.86 | 10.0±3.83 | 11.4±3.90 | 10.1±3.37 | | 10.5±3.55 |
| Week 52/ET^b^ | 6.0 (–) | 7.6±1.69 | 7.9±2.15 | 9.5±3.30 | 9.4±3.30 | | 8.9±3.04 |
| Sleep-onset delay |  |  |  |  |  | |  |
| Baseline^a^ | 3.0 (–) | 1.9±0.79 | 2.0±0.86 | 2.0±0.82 | 1.8±0.82 | | 1.9±0.82 |
| Week 52/ET^b^ | 1.0 (–) | 1.4±0.50 | 1.5±0.62 | 1.7±0.81 | 1.6±0.65 | | 1.6±0.67 |
| Sleep duration |  |  |  |  |  | |  |
| Baseline^a^ | 8.0 (–) | 4.3±1.14 | 4.2±1.63 | 4.5±1.65 | 4.3±1.59 | | 4.4±1.59 |
| Week 52/ET^b^ | 3.0 (–) | 3.6±0.92 | 3.3±0.83 | 4.0±1.41 | 3.9±1.37 | | 3.8±1.26 |
| Sleep anxiety |  |  |  |  |  | |  |
| Baseline^a,c^ | 11.0 (–) | 6.7±2.02 | 6.6±2.13 | 7.0±2.39 | 6.5±2.50 | | 6.7±2.36 |
| Week 52/ET^d^ | 4.0 (–) | 4.5±1.04 | 5.0±1.24 | 5.6±2.15 | 6.0±2.39 | | 5.5±2.09 |
| Night wakings |  |  |  |  |  | |  |
| Baseline^a,c^ | 5.0 (–) | 5.3±1.50 | 4.2±1.47 | 5.7±1.83 | 5.0±1.98 | | 5.0±1.85 |
| Week 52/ET^b^ | 3.0 (–) | 3.6±1.03 | 3.4±0.78 | 4.6±1.80 | 4.5±1.71 | | 4.2±1.59 |
| Parasomnias |  |  |  |  |  | |  |
| Baseline^a,c^ | 10.0 (–) | 10.3±3.14 | 9.9±2.46 | 10.3±2.65 | 9.8±2.48 | | 10.0±2.56 |
| Week 52/ET^e^ | 9.0 (–) | 8.4±1.58 | 8.1±0.83 | 8.3±1.61 | 8.9±1.68 | | 8.6±1.54 |
| Sleep-disordered breathing |  |  |  |  |  | |  |
| Baseline^a,c^ | 4.0 (–) | 3.8±0.97 | 3.4±0.92 | 3.8±1.62 | 3.8±1.25 | | 3.7±1.26 |
| Week 52/ET^b^ | 3.0 (–) | 3.4±0.67 | 3.2±0.73 | 3.2±0.49 | 3.5±0.84 | | 3.4±0.74 |
| Daytime sleepiness |  |  |  |  |  | |  |
| Baseline^a,c^ | 21.0 (–) | 10.4±2.54 | 11.6±4.68 | 11.4±3.55 | 11.8±3.49 | | 11.6±3.74 |
| Week 52/ET^b^ | 7.0 (–) | 9.3±3.10 | 9.7±3.09 | 10.6±4.94 | 10.3±3.59 | | 10.1±3.78 |

LDX=lisdexamfetamine dimesylate; week 52/ET=data from protocol-defined last treatment study visit or early termination visit.

^a^Baseline is defined as the baseline value from the antecedent study (Ph 2 Study [NCT02402166]; Ph 3 study [NCT03260205]) for antecedent participants, or the last observation before the first dose of investigational product for directly enrolled participants.

^b^n=11 (10 mg), n=18 (15 mg), n=23 (20 mg), n=49 (30 mg), n=102 (total).

^c^n=52 (30 mg), n=112 (total).

^d^n=11 (10 mg), n=18 (15 mg), n=23 (20 mg), n=48 (30 mg), n=101 (total).

^e^n=10 (10 mg), n=18 (15 mg), n=23 (20 mg), n=47 (30 mg), n=99 (total).
