# Supplementary material for: A Long-Term, Open-Label Safety and Tolerability Study of Lisdexamfetamine Dimesylate in Children Aged 4–5 Years with Attention-Deficit/Hyperactivity Disorder
Source: J Child Adolesc Psychopharmacol. 2022 Mar 15;32(2):98–106. doi: 10.1089/cap.2021.0138 (PMC8971990; doi:10.1089/cap.2021.0138)
Supplement: Supplemental data [file Suppl_TableS2.docx]

# Supplementary Table 2. Daytime Napping, Nighttime Sleep, and Time to Fall Asleep Based on Sleep Diaries

| **Parameter, mean ± SD** | **5 mg**  **(n=1)** | **10 mg**  **(n=12)** | **15 mg**  **(n=21)** | **20 mg**  **(n=26)** | **30 mg**  **(n=53)** | **Total**  **(N=113)** |
| --- | --- | --- | --- | --- | --- | --- |
| Daily daytime napping, hours |  |  |  |  |  |  |
| Baseline^a,b^ | 2.5 (–) | 0.2±0.22 | 0.3±0.41 | 0.4±0.60 | 0.4±0.59 | 0.4±0.57 |
| Week 52/ET^c^ | 0.0 (–) | 0.0±0.05 | 0.2±0.39 | 0.1±0.39 | 0.2±0.29 | 0.2±0.32 |
| Daily nighttime sleep, hours |  |  |  |  |  |  |
| Baseline^a,d^ | 6.6 (–) | 9.9±0.63 | 9.1±2.57 | 9.5±1.03 | 9.0±2.41 | 9.2±2.07 |
| Week 52/ET^c^ | 10.1 (–) | 10.2±0.58 | 10.1±0.81 | 9.8±0.78 | 10.0±0.64 | 10.0±0.70 |
| Daily time to fall asleep, hours |  |  |  |  |  |  |
| Baseline^a,d^ | 1.9 (–) | 0.5±0.36 | 1.1±2.14 | 0.7±0.49 | 1.1±1.95 | 0.9±1.64 |
| Week 52/ET^c^ | 0.2 (–) | 0.3±0.20 | 0.4±0.38 | 0.6±0.47 | 0.4±0.32 | 0.4±0.37 |

LDX=lisdexamfetamine dimesylate; week 52/ET=data from protocol-defined last treatment study visit or early termination visit.

^a^Baseline is defined as the baseline value from the antecedent study (Ph 2 Study [NCT02402166]; Ph 3 study [NCT03260205]) for antecedent participants, or the last observation before the first dose of investigational product for directly enrolled participants.

^b^n=20 (15 mg), n=25 (20 mg), n=51 (30 mg), n=109 (total).

^c^n=8 (10 mg), n=16 (15 mg), n=23 (20 mg), n=43 (30 mg), n=91 (total).

^d^n=20 (15 mg), n=25 (20 mg), n=50 (30 mg), n=108 (total).
